# Breathing to younger skin: ‘reversing the molecular mechanism of skin aging with yoga’

**DOI:** 10.4155/fsoa-2016-0015

**Published:** 2016-05-09

**Authors:** Kavita Beri

**Affiliations:** 1 Center For Dermal Research, Department of Biomaterials, Rutgers – The State University of New Jersey, Life Sciences Building, 145 Bevier Road, Piscataway, NJ 08854, USA

**Keywords:** breathing, epidermis, molecular, pranayama, skin aging, yoga

In recent times, the practice of yoga has taken a more mainstream popularity in a fast-paced life. This ancient tradition that has resurfaced has many hidden values, and challenges modern day science to undergo new research and increase understanding of its impact on the body. This article is a commentary aiming to correlate and extrapolate recent evidence-based research on some of the known molecular benefits of yoga, and its possible correlation with skin aging. Yoga is a 5000-year-old ancient Indian way of life, which includes changes in mental attitude, diet and the practice of specific techniques such as yoga asanas (postures), breathing practices (pranayama) and meditation to attain the highest level of consciousness [1]. The increasingly wide use of these practices has triggered numerous research studies, especially in recent decades, which suggest that yogic/meditative practices have significant positive effects on the mind–body system and thereby can have an effect on overall improved health and quality of life [2,3]. Skin appearance is a primary indicator of age and, like most organs of the body, undergoes changes over the passage of time [4]. The molecular and cellular process of skin aging is similar to that occurring in most internal organs and involves slow deterioration in tissue function. The stratum corneum layer might remained unchanged but the overall thickness of the epidermis and dermis is affected in aging skin, with a noted flattening of the dermoepidermal junctions [5].

## Overview of cellular & molecular skin aging

There is a decreased proliferative capacity of skin cells taken from old donors versus younger individuals – this has been described in a number of studies with keratinocytes [6] and fibroblasts [7]. At the end of their replicative life span, cellular senescence is described as the arrest of cells growth at G1 phase [5]. Senescent skin fibroblast have increased expression of *IL-1* [8] and of the *EGF*-like cytokine heregulin, which modulates the growth and differentiation of breast and other epithelial cells [9]. Thus, it can be said that the balance between growth and differentiation of overlying epithelial cells is dependent on senescent stromal cells. Cumulative oxidative damage has been shown to play a key role in molecular dysfunction [10]. For the skin, with its high exposure to environmental agents such as ultraviolet radiation and ozone, cumulative oxidative damage is very relative [5]. The reactive species lead to damage of lipids, proteins and DNA and also influence cellular senescence [11]. Once the cells are forced to enter a senescent state, with even low doses of H_2_O_2_, the proliferative and differentiation changes described above are seen [11]. There is a reduction of antioxidant enzymes in skin with age including Cu, Zn-superoxide dismutase (SOD), catalase and glutathione peroxidase [12]. For human skin fibroblasts, senescence results in reduced collagen and increased *MMP-1* production. Aerobic and anaerobic energy metabolism with the mitochondria generate reactive oxygen species that oxidize cellular constituents thereby impairing cell function [13]. The aged phenotype results from irreversible cellular damage from the oxidative stress [14]. Photoaging can be considered as accelerated chronological aging because UV irradiation acutely induces collagen-degrading *MMP* activities and suppresses collagen production [15–17]. Thus, there is collagen degradation noted with UV irradiation leading to chronological aging.

## Recent evidence-based, biomolecular effects of yoga

The effects of both yogic postures (asanas) and breathing techniques (pranayama) have been shown to be correlated with a downregulation of the hypothalamic–pituitary–adrenal axis and the sympathetic nervous system, both of which are known to be overactivated by a western lifestyle [18,19]. One study investigated possible changes in gene expression induced by the practice of S Kriya and its associated practices (SK&P), which is a comprehensive yoga program including breathing and meditative exercises [20]. The study included 42 SK&P practitioners and 42 normal healthy controls. RNA was isolated from PBMCs (polymorphonuclear cells), and subjected to RT-PCR analysis with a focus on genes involved in oxidative stress, DNA damage, cell cycle control, aging and apoptosis. In parallel, blood was drawn and subjected to assays to determine glutathione peroxidase levels, SOD activity and glutathione levels. Consistent with a previous study [21], glutathione peroxidase and superoxide dismutase activities and glutathione levels were higher in SK&P practitioners compared with controls. In keeping with these data, glutathione S-transferase mRNA expression was significantly higher in the SK&P group compared with controls [20]. A similar increase in the SK&P group was observed in the antioxidant genes Cu-Zn and Mn *SOD*, glutathione peroxidase and catalase [19]. In addition, expression of the antiapoptotic gene *COX-2* and stress response gene *HSP-70* were significantly increased in the SK&P group. The antiapoptotic gene *BCL-2* and aging-related gene *TERT* displayed an increasing trend in the SK&P practitioners, but were not significantly altered. Based on these findings the authors suggested that SK&P might trigger an improved antioxidant status, at least in part owing to changes in the expression of the relevant genes, which may translate into better response to environmental stress [19]. The increased levels of *COX-2* and *BCL-2* expression were suggested to prolong the lifespan of PBMCs via inhibition of apoptosis, which may lead to better immune functioning and protection from disease [20]. The analysis of a larger number of genes in the respective pathways is warranted. Changes in the population of NK cells were observed in response to SK&P – the cell population continued to increase over time with continued practice of SK&P [21,22]. A study exploring the effects of yoga practice on inflammatory markers after moderate and strenuous exercise showed the yoga group to exhibit reduced inflammatory response as evidenced by lower levels of *TNF-α* and *IL-6* [23].

## Impact on tissue glycation through yoga

Advanced glycation end products (AGE) have recently been shown to play a role in tissue aging [24]. Their precise role in aging skin is still not clearly understood. As aging happens in each cell of the body, evidence shows an improvement in tissue functioning and improved glucose control with the practice of yoga. Tissue glycation and peripheral glucose control can be influenced by regeneration of cells in the liver and pancreas [25]. With side stretching and different side body opening postures, it is believed that there is a mechanical tissue stimulation resulting in increased utilization and metabolism of glucose in peripheral tissues, liver and adipose tissues through enzymatic process [25,26]. With poses that build strength and flexibility in the muscles, enhanced blood flow and insulin receptor expression on the muscles result in increased glucose uptake and hence reduced blood sugar [24,27]. The improvement in lipid levels after yoga could be due to increased hepatic lipase and lipoprotein lipase at the cellular level, which affects the metabolism of lipoprotein and thus increases uptake of triglycerides by adipose tissues [19,22]. There is an overall improvement in metabolic functioning of the cells and tissue.

## The neurophysiologic effects & antiaging through de-stressing

A known phenomenon is increased wrinkles through constant frowning [4]. Stress and anxiety have become a normal part of modern existence. There is a need for individuals to adopt a stress-free routine in their schedule. Yoga provides what is often described as a ‘spiritual connection’ and aids in relaxation of the mind and body, reducing and improving overall levels of stress [19]. The effects of yoga on the hypothalamic–pituitary–adrenal–cortisol axis play an important role in helping patients with depression and post-traumatic stress disorder [22].

## Conclusion: is yoga the path of discovering a ‘fountain of youth’

The practice of yoga with a ‘yogic attitude’ (patience, persistent practice and realizing the self) one should be able to reduce laziness, anger, delusion and desire for being different or better than others [20,21]. Through expressing improved self-image and ahimsa (nonviolence to the self) there is an overall improvement in the quality of life and mood of the individual [2]. The evidences supporting the antioxidant cellular and tissue effects of yoga discovered in the recent decade are intriguing and warrant more studies with a larger population base [19]. Certain yoga postures such as twisting, binding and certain back-bend positions according to ayurveda are suggested to help proper flow of the prana (‘life force’) and help detox the body [27]; scientific studies in relation to these theories will shed more light on the esoteric nature of yoga science. Can the antioxidant molecular and cellular benefits of the practice of yoga impact the aging of skin cells? The interesting correlation of insulin regulation and glucose control can be translated to improvement and maybe reversal of the effects on AGE protein accumulation in the body tissue. More biochemical research studies will aid in further exploring this interesting connection. In the above commentary, we hope to draw an extrapolation between the known cellular and molecular changes with yoga and aging skin cells. The author hopes to inspire future research, taking this age-old practice and correlating it to the processes of cellular senescence and dysfunction. For a dynamic organ like the skin, research could also focus on phenotypic and histologic changes resulting from regular yoga and meditative practices in different patient populations, with a view to incorporating a yoga approach into holistic antiaging cosmetic skin rejuvenation.
